# Experimental Verification of Geometric Changes Caused by the Release of Residual Stresses for Large-Scale Welded Frames

**DOI:** 10.3390/ma17102389

**Published:** 2024-05-16

**Authors:** Michał Wieczorowski, Michał Jakubowicz, Lidia Marciniak-Podsadna, Bartosz Gapiński, Roman Barczewski, Bartosz Jakubek, Filip Rogiewicz, Czesław Jermak, Rehan Khan

**Affiliations:** 1 Division of Metrology and Measurement Systems, Institute of Mechanical Technology, Faculty of Mechanical Engineering, Poznan University of Technology, 5 M. Skłodowska-Curie Square, 60-965 Poznan, Poland; michal.jakubowicz@put.poznan.pl (M.J.); lidia.marciniak-podsadna@put.poznan.pl (L.M.-P.); bartosz.gapinski@put.poznan.pl (B.G.); 2 Division of Vibroacoustics and Diagnostics of Systems, Institute of Applied Mechanics, Faculty of Mechanical Engineering, Poznan University of Technology, 5 M. Skłodowska-Curie Square, 60-965 Poznan, Poland; roman.barczewski@put.poznan.pl (R.B.); bartosz.jakubek@put.poznan.pl (B.J.); 3 PROTiM Sp. z o. o., 8D Grzybowa Street, 62-081 Wysogotowo, Poland; f.rogiewicz@protim.pl (F.R.); c.jermak@protim.pl (C.J.); 4 Department of Mechanical Engineering, College of Electrical and Mechanical Engineering, National University of Sciences and Technology, Islamabad 44000, Pakistan; mrehan.khan@ceme.nust.edu.pk

**Keywords:** coordinate measuring technique, photogrammetry, vibratory stress relief, residual stresses

## Abstract

This paper presents geometric analyses of welded frames after free relaxing and vibratory stress relief (VSR). The tested frames were components of a prototype packaging machine. Two types of relaxation were carried out to remove stresses introduced as a result of the welding process. One of the frames was subjected to free relaxation, while the other one was subjected to accelerated vibration relaxation. Detection of the frame geometry changes was performed using a photogrammetric system. In addition, an evaluation of the geometry change was conducted for fifteen variants of a steel frame support. A comparative analysis of the geometric deviations of the frames after free and vibratory stress relief confirmed the assumption that the frame post vibration stress relief better reproduces the nominal dimensions. Nevertheless, it should be emphasized that after vibratory stress relief, the frame is not subject to further deformation, which is a desirable effect. In the case of free relaxing, the frame undergoes dimensional changes in a random manner. In summary, carrying out accelerated vibratory stress relief allows for control of spontaneous dimensional changes in the designed frame of a packaging machine resulting from spontaneous relaxation of stresses arising from the welding process. The shortening of the relaxation process of the welded frame is also an unquestionable advantage.

## 1. Introduction

Residual stresses occur in metal structures during many technological processes. They can arise as a result of body force or surface stress, under the action of temperature, or are as result of phase changes occurring in the material [1,2]. The phenomenon of residual stresses is undesirable because it negatively affects the dimensional stability of the structure, causing it to deform over a long period of time as a result of the spontaneous removal of these stresses, and it also increases susceptibility to cracking and stress corrosion cracking [3,4]. Wang et al. [5] presented the thesis that residual stresses do not always limit the functionality of the material. They described [5] that the peening process aims to improve the fatigue properties of the material by introducing residual compressive stress at the surface of the specimen.

One of the technological processes affecting residual stresses in steel structures is welding. This is a process during which a welded component is subjected to complex thermal processes, which lead to structural changes. The ultimate effect of this process is the formation of stresses, the distributions of which vary greatly both in direction and value. This, in turn, is the cause of geometric deformations [2].

One way to reduce over time the stresses present in a component after the welding process until they are completely removed is relaxation [2]. Several relaxation methods can be distinguished: annealing relaxation [6,7], ultrasonic relaxation [8], rolling [9], the explosive method [10] and vibratory stress relief [11,12]. The authors of the paper [13] also presented a hybrid method that combines the advantages of thermal vibration stress relief (TVSR).

The method of residual stress relaxation by vibratory stress relief (VSR) is invaluable in many cases due to its short duration. Vibratory stress relief (VSR) consists of the accelerated technical degradation of machine and structural components by subjecting them to mainly resonant vibrations. By forcing various forms of resonant vibration throughout the volume of the vibrated component, a significant reduction in peak internal (weld) stresses can be produced [14].

Application of vibratory loading to reduction of residual stress levels in mechanical components may be a potential alternative to some thermal annealing processes [15]. The use of the vibratory stress relief (VSR) process has been limited in use due to a poor understanding of the process, as indicated by Hassan [15]. Another paper [16] presents various variants of vibratory stress relief (VSR) annealing. A broad overview of the advantages, disadvantages and applicability in various industrial conditions of VSR variants such as resonant VSR, sub-harmonic VSR and modal sub-resonant VSR is presented in [17,18]. An applied use of vibratory stress relief for the relaxation of welded bridge structural components is presented in [19], while in [20], VSR is used for the relaxation of residual stresses after the rolling process as an alternative to the much more time-consuming temperature method.

In another application example, the vibratory stress relief (VSR) procedure was used to reduce welding-induced residual stresses in the rails of a magnetic levitation transport system (MAGLEV) [21]. In [22], it is shown that the fatigue life of thermally relaxed samples decreased by 43%, while samples subjected to vibration showed an increase from 17% to 30%.

Stress relaxation techniques should be performed immediately after the technological process, as otherwise, free relaxation may occur (i.e., the stresses will be removed spontaneously). However, it should be borne in mind that the relaxation process, whether free or forced, causes deformation of the component, and these deformations can negatively affect the functionality of the finished product.

Therefore, an important issue during the stress removal process is the dimensional and shape control of relaxed objects. The propagation of geometric deformations during and after the vibratory stress relief process has not yet been described in detail in the literature. Knowledge of the described relationships is important in determining the direction and value of geometric deformation, which clearly affect the final functionality of the product.

Measuring methods, due to the contact of the measuring instrument with the tested object, can be divided into contact and non-contact [23,24]. Contact methods include traditional measurements with CMMs, where the measurement is realized by the point of contact between the tip of the measuring head (usually spherical) and the surface to be measured [25]. This method has certain limitations related to the size and shape of the object being tested. The second group of measurement methods are non-contact methods, in which there is no contact between the measuring instrument and the object being measured [26,27]. This allows for accurate measurements of objects with complex shapes, of both small and large size [28,29,30,31], as well as those made of flexible materials such as sponges, foams, or plastics [32,33] or made by additive technologies [34,35,36]; the same applies to metal elements manufactured by wire arc additive manufacturing [37]. For such large components as the welded frame under study, the use of a photogrammetric method is both reasonable and preferable.

Three-dimensional scanning technology is increasingly used in industry to control the dimensions of objects with different spatial configurations. The wide application and development prospects of 3D scanning technology are presented in article [38]. Quality requirements for manufactured products have enforced the use of a method to analyze them in terms of shape and dimensions. Robotized measuring systems using optical scanners, in addition to a large number of measuring points and the lack of need for physical contact with the part being measured, also provide speed and repeatability of measurements [39]. This technology also has growing potential for measuring at smaller scales [40]. Thus, the use of 3D scanning technology is becoming an integral condition for ensuring that manufactured products have the required quality and for eliminating errors on every stage of the production process.

The main objective of the research presented in this article was to evaluate the dimensional changes in welded frames subjected to spontaneous, free relaxation (frame No. 1) and rapid relaxation using a vibratory stress relief method (frame No. 2). The study also made it possible to assess the quality of frame assembly using welded joints from the perspective of dimensional and shape accuracy. The research components are frames of a prototype packaging machine designed and built by PROTiM company.

**Figure 1:**
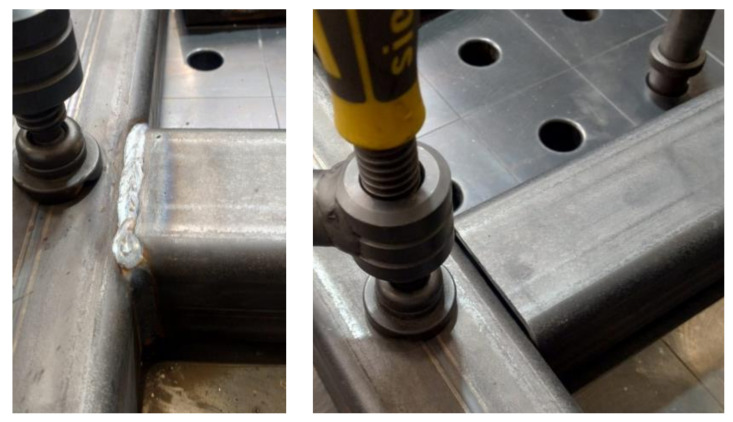
An example of a weld connecting two structural components of a packaging machine (frame No. 1).

The objects of the study are the steel frames that form the basis of the test stand. The analyzed frames are formed in the joining process by welding closed sections of 120 × 80 × 8 made of material S235JRH/S275J0H (Figure 1). The welding method used is gas metal arc welding (GMAW) (MIG C340 Pro welder) argon and CO_2_ shielded welding in a ratio of 80–20%. Gas metal arc welding (GMAW), known by its variants metal inert gas (MIG) and metal active gas (MAG), is a welding technique in which an electric arc is generated between a consumable MIG wire electrode and the metal being worked on. This arc heats the metal, leading to their fusion (melting and joining). In addition to the wire electrode, a shielding gas is supplied through the welding gun to protect the process from atmospheric impurities. Before laying the main weld, the parts are initially joined with short welds. The welding parameters are as follows: 180–190 A, wire MC2K300MSPRE, diameter 1 mm, and weld laying speed about 10 mm/s.

Engineering science provides a theoretical basis for the statement that residual stresses influence the deformation of components after machining and welding. Based on this knowledge, the authors decided to experimentally test how large this influence is in the case of a large-scale prototype part. They used an accurate 3D laser scanning method to obtain a model of the part immediately after machining, which forms the basis for evaluating the two methods of residual stress release, and then experimentally determined the effect of the method and time of release on the final deformation of the part. The comparative method developed allowed the validity of alternative relaxation methods for the welded frames of the prototype packaging machine to be established.

In addition, the effect of the number and arrangement of support points on the welded frame’s geometric deformation was analyzed. Due to its dimensions, only a selected part of the frame, which is the basis of the test stand, was adopted for analysis. The frame intended for testing in the following part of the work was named frame No. 3. During the measurement process, temperature changes were recorded at selected points of the tested objects.

## 2. Materials and Methods

### 2.1. Process of Vibratory Stress Relief of Welded Frames

Vibratory stress relief consists of subjecting the mechanical structure, in this case the welded frame, to forced vibration. The forcing is carried out at resonant frequencies corresponding to different forms (modes) of natural vibration of the frame. As a result of the forced vibration of the mechanical structure, residual stresses are relaxed, which prevents further deformation and/or cracking of the frame once it is installed in the target machine during its service life.

**Figure 2:**
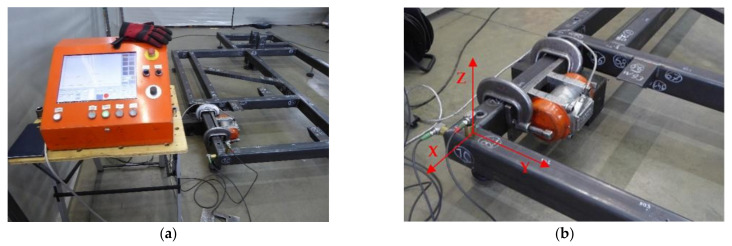
Vibratory stress relief instrumentation kit: control and monitoring unit (**a**); eccentric vibrator mounted on frame and vibration acceleration transducers monitoring the vibration process (**b**).

Figure 1 shows the vibratory stress relief kit used in the study. It consisted of a control and monitoring unit from Wibropol and a WC2020 eccentric vibrator (WIBROPOL Marek Majewski, Promienko, Poland) mounted on the frame being relaxed. The location at which the vibrator is attached is crucial. It should ensure that the structure can be stimulated to vibrate with multiple modes. Thus, the vibrator should be attached preferably in places where the nodal points of individual vibration forms are not located. The adopted method of attaching the vibrator (its orientation) with respect to the frame made it possible to effectively excite the structure to vibration in the vertical Z and horizontal Y directions (Figure 2).

**Figure 3:**
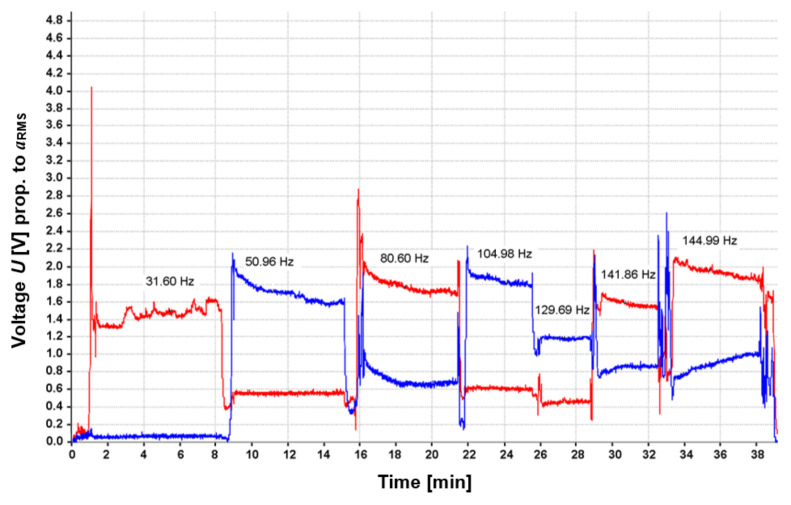
Course of sequential vibratory stress relief of the frame.

**Figure 4:**
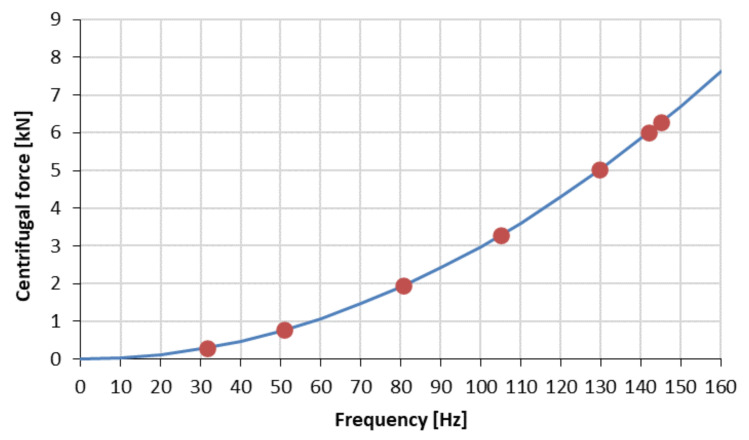
Dependence of the centrifugal force generated by an eccentric vibrator on its rotational frequency.

Information on the RMS values of vibration accelerations from two accelerometers mounted vertically and horizontally on the frame was acquired from the control panel. The vibration transducers were mounted near the vibrator in the y–z plane, which was the plane of excitation of the frame to vibration. The diagram in Figure 3 shows the course of the sequential vibratory stress relief process. The course marked in red corresponds to vibration accelerations recorded in the vertical z-direction, and blue indicates vibrations in the y-direction. The values of the vertical axis of the graph express voltage [V] proportional to the RMS values of vibration accelerations recorded by accelerometers. On the horizontal axis, time is presented in seconds. Table 1 lists the parameters of sequentially conducted vibratory stress relief. Meanwhile, the dependence of the centrifugal force generated in the vibration stress relief process on the frequency of the eccentric vibrator shaft is provided in Figure 4.

### 2.2. Analysis of Geometric Features Using Spatial Photogrammetry

**Figure 5:**
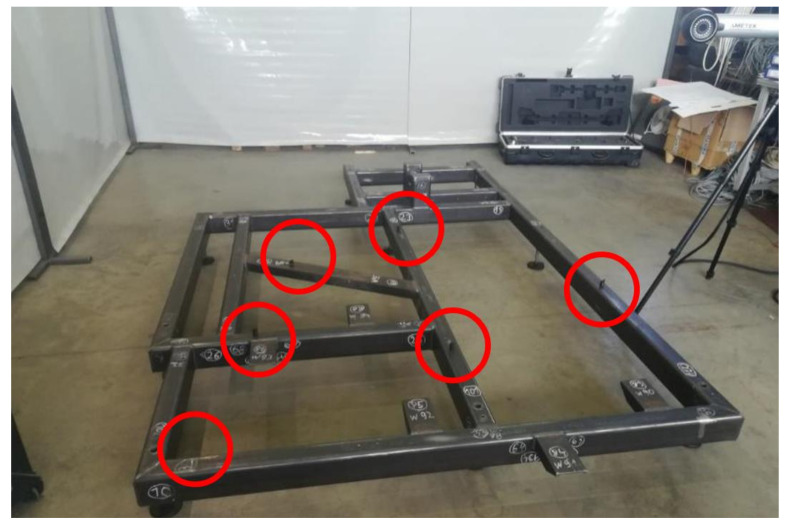
Example of the location of reference points on the tested frame No. 1.

To measure the geometric features of the steel frames, a laser scanner from Creaform was used, working with VXelements 6.2 software (VXelements 6.2, Creaform Inc., Lévis, QC, Canada). It is a fast and accurate scanner that can be used on production lines for automatic or manual 3D inspection of parts. The scanner has many applications, such as product quality control and evaluation of 3D models’ compatibility with the nominal model, and enables reverse engineering, 3D scanning to CAD, CAD modeling, or finite element analysis. Analysis of the scans obtained was carried out using GOM Inspect software. Prior to taking measurements with a 3D scanner, reference points were attached to the frame. These points were placed randomly with the assumption that the scanner would “see” at least four in each shot (Figure 5).

The presented tests of welded frames (frame No. 1 and frame No. 2) were carried out at the headquarters of PROTiM (Wysogotowo, Poland). Tests on the impact of the location of support points (frame No. 3) were carried out in the research laboratory located at the Department of Metrology and Measurement Systems of the Poznan University of Technology (Poznań, Poland).

**Figure 6:**
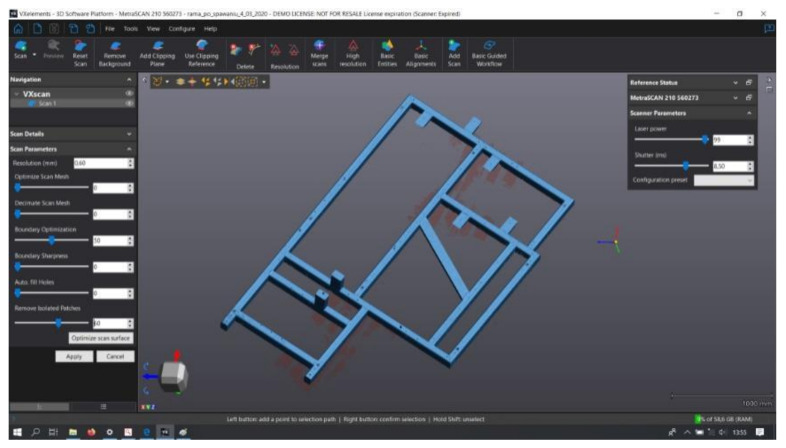
Example of a point cloud tested frame No. 1 (VXelements 6.2 software).

As a result of the measurements, measurement data obtained in the form of a point cloud were subjected to further processing and optimization. Point clouds corresponding to the measurement data were polygonized. This process consists of building a model in the form of a triangle grid, with vertices located at individual points in the collection, and it generates files in STL format and creates 3D models of measured objects. The scanning of each object produces many individual scans, which must be automatically combined using an intelligent algorithm based on surface matching and on the combination of reference points to obtain the most complete model possible. An example of a measurement point cloud of a scanned welded frame is shown in Figure 6.

**Figure 7:**
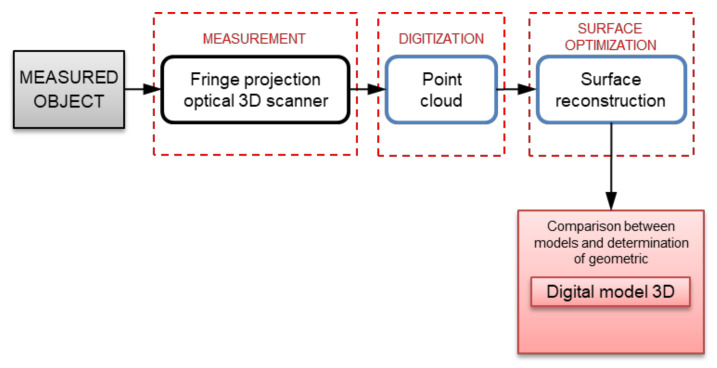
Stages of analysis of the obtained measurement results.

The next steps of the measurement and data analysis procedure of the obtained point cloud are presented below in the form of a graphic diagram (Figure 7).

The following tasks related to the determination of geometric characteristics were carried out for different states of the frame:

- Scanning of a welded steel frame (frame No. 1)—measurement immediately after welding.
- Scanning of a welded steel frame (frame No. 1)—measurement after free relaxation.
- Scanning of a welded steel frame (frame No. 2)—measurement before vibratory stress relief (measurement immediately after welding).
- Scanning of a welded steel frame (frame No. 2)—measurement after vibratory stress relief.
- Measurement of temperature distribution at the characteristic points of the tested frames during the measurement.

The comparison of elements is presented in the form of a colorful map, where deviations at the inspection points are represented by a color scale. One of the criteria for properly measuring of welded structure geometry is proper location of their support points.

The construction under study is the base of the packaging machine and has a large size, making it necessary to carefully consider the support points. Their location should not generate an additional deflection of the whole structure. They were defined to correspond to the support points of the final structure. In order to verify assumptions, an analysis of a welded steel frame with different support points (with 15 variants) was carried out (frame No. 3). The study was conducted for a welded frame made of the same profiles as a vibration relaxed frame, which is a section of the original final frame.

**Figure 8:**
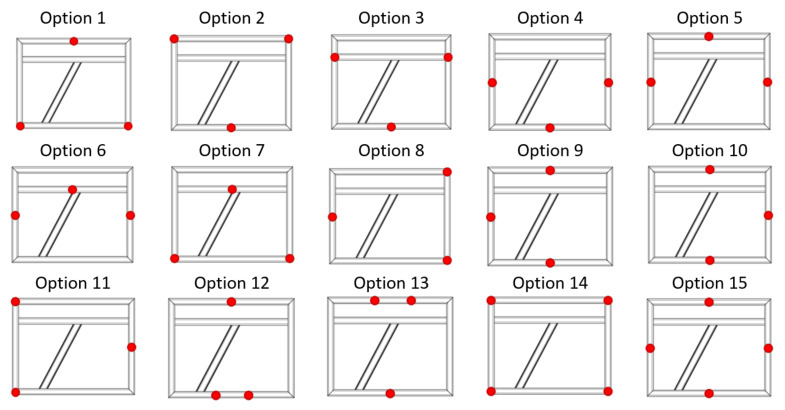
Example of the location of reference points on the tested frame No. 3.

Figure 8 shows the schematics of designed frame for analysis of the effect of support points’ distribution on the results of the measurement, along with the marked support points.

## 3. Results and Discussion

### 3.1. Analyses of the Obtained Results

**Figure 9:**
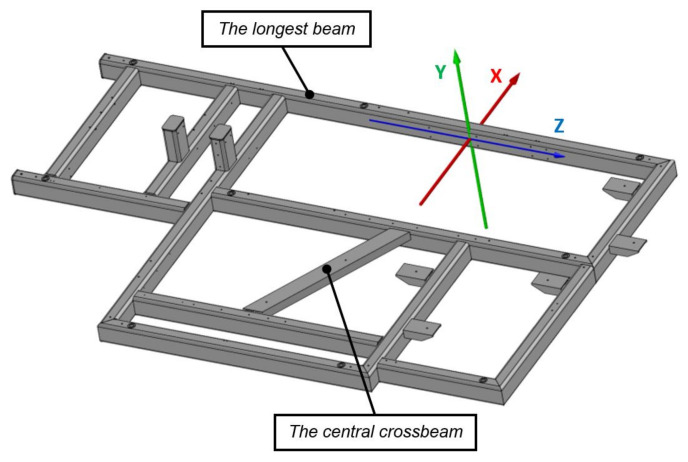
CAD model of the tested frame with its coordinate system.

The analysis focused on the geometric characteristics of the welded frames, examining them both after free relaxation and after vibratory stress relief. The study involved comparing the geometry of two frames, designated frame 1 and frame 2, at various stages of the welding process with the corresponding CAD models (Autodesk Inventor software 2023, Autodesk, San Rafael, CA, USA). For each comparison, two methods were used: the best-fit method and the 3-2-1 method. The CAD model, which is the basis for the construction of the tested components and is a reference for comparative analysis, is shown in Figure 9.

Under the best-fit method, colorful deviation maps were generated to illustrate maximum and minimum deviations in different areas, accompanied by *Y*-axis inspection sections highlighting maximum and minimum deviation values in individual areas. Cross-sections in the *Z*-axis were also examined, specifically focusing on the front plane of the frame to indicate geometrical deviations.

Similarly, the 3-2-1 method was applied to analyze deviations, employing deviation maps, *Y*-axis inspection sections, and cross-sections on the *Z*-axis.

Additionally, the flatness of the frame face was measured as part of the comprehensive analysis of the welded frames. Overall, these meticulous comparisons and analyses provided valuable information on the geometric changes that occur in welded frames during and after the welding process, contributing to a deeper understanding of their structural integrity and quality.

### 3.2. Comparison of the Geometry of Frame 1 Immediately after Welding with the CAD Model

**Figure 10:**
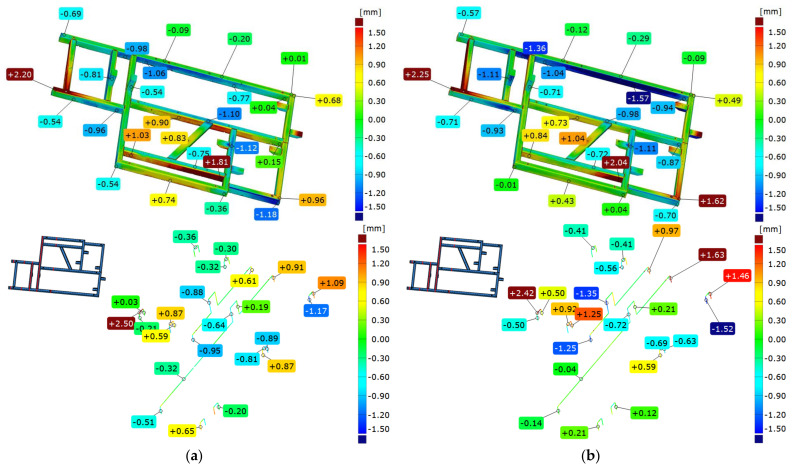
Example of comparison geometry of frame 1 immediately after welding (**a**) and after free relaxation (**b**) with the CAD model.

The geometry analysis was carried out in two alignments. The first is based on aligning the mesh representing measured element with a nominal spatial model in the form of a CAD model, according to the best-fit (based on the least-squares method). In this comparison, digital representation of the real element is aligned with the CAD model in such a way that the sum of deviations squares between corresponding points tends to be 0. On the basis of this alignment, a colorful map of deviations is presented. The distribution of values shows how complex the nature of the deformation is between the real and the nominal element. The spread of obtained values reaches nearly 4.7 mm, while the flatness of the upper frame surface is 3.30 mm. Figure 10a shows an example of a colorful deviation map for the comparison of the geometry of frame 1 immediately after welding with the CAD model.

Analyzing cross-sections parallel to the *Y*-axis, it can be seen that the displacement of mounting posts is −0.88 mm for the former and −0.64 mm for the latter on the frame just after welding. Meanwhile, after free relaxation, the displacement in the corresponding points is −1.35 mm for the former and −0.72 mm for the latter.

The entire analysis was repeated using the second concept of alignment—the 3-2-1 method. In this method, the alignment of the CAD model and the measured mesh representing a real element was carried out using geometric elements receiving successive degrees of freedom of the element (plane—3 degrees of freedom, line—2, point—1). Here, both the values and distribution of deviations take on a different character. The spread of deviation is 5.6 mm, nearly 1 mm more than in the case of the first concept. The diagonal beam in both cases shows convexity, and mounting posts are offset in the negative direction; when averaging the fit (best-fit), this offset has a larger absolute value. In both alignments, the largest value of deviation at the face occurs at the same place—that is, at the longest edge of the frame. When analyzing deformations in XZ plane, it is observed that flatness of the element changed dramatically. Deviations in bottom right corner changed from 0.96 mm to 1.62 mm, while in the other corner of the frame, changes in deviations were similar to those registered straight after welding.

### 3.3. Comparison of Frame 1’s Geometry after Free Relaxation with the CAD Model

The next analysis shows the comparison of frame 1 after free relaxation with the nominal CAD model. The analysis methodology presented in the previous section is retained. For the best-fit alignment, the colorful deviation map shows two characteristic areas on the face of the frame where deviations have the largest values. These values show the propagation of frame deformation due to stress relaxation. The sections also show further displacement of the mounting posts (−1.35 mm and −0.72 mm, respectively). Also noteworthy is the deviation of the longest beam location, which is +1.63 mm, showing an increase in convexity in this area. Figure 10b shows a colorful deviation map for the comparison of the geometry of frame 1 after free relaxation with the CAD.

### 3.4. Comparison of the Geometry of Frame 1 Immediately after Welding and after Free Relaxation

**Figure 11:**
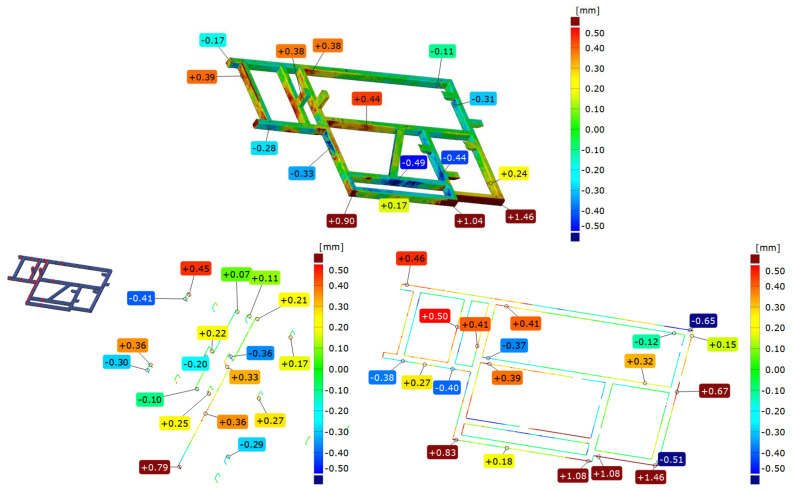
Example of comparison the geometry of frame 1 immediately after welding and after free relaxation (best-fit alignment).

The ambiguity of the results obtained, as well as some difficulties in correctly interpreting the changes in deviations when comparing the measured frame with the CAD model both immediately after welding and after free relaxation, led to another analysis, which consisted of comparing two frame scans with each other. Figure 11 illustrates a colorful deviation map exemplifying the comparison of frame 1’s geometry immediately after welding and following a period of free relaxation (best-fit alignment).

The starting element, taken as a nominal one, was established as the frame scan obtained immediately after welding. A scan of the same frame after free relaxation was then aligned to it and the results compared. According to the assumptions, the analysis was carried out in two coordinate systems (using two alignments)—best-fit and the 3-2-1 method. Analyzing the results of the comparison according to the best-fit method, one can see deviation values of up to 1.5 mm, which shows the order of magnitude of deformation with such a large frame resulting from the release of residual welding stresses. Some reciprocal torsion between the components can be seen here, as shown by deviations in the longest beam of the frame. On the opposite beam, on the other hand, concavity is observed; this indicates that as a result of relaxation, the central part of this beam has deformed in the positive direction less than in the node area of the structure, where the deviation values are 4–5 times higher.

Using 3-2-1 alignment, a similar trend of concavity is noticed, but with different proportions. Here, the deviation on the central part of the beam and on nodes changes by 2–3 times. With this method of aligning, the torsion of the system is significantly reduced and amounts to 0.2 mm, which, for a welded element of such large dimensions, does not seem to be significant. The frontal section shows a displacement of the beams reaching +1.46 mm, which suggests that special attention should be given to this particular area when assembling subsequent elements of the device.

### 3.5. Comparison of Frame 2’s Geometry Immediately after Welding with the CAD Model

**Figure 12:**
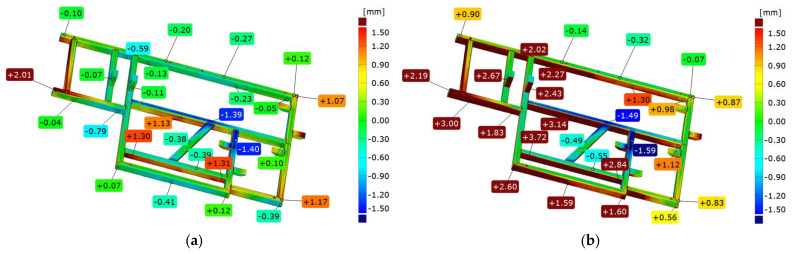
Example of comparison of the geometry of frame 2 immediately after welding with the CAD model according to two strategies of alignment: best-fit (**a**) and 3-2-1 (**b**).

According to the geometric specifications, a second sample, called “frame 2”, was prepared. It was subjected to an analogous analysis as frame 1. In the first step, frame 2 was compared immediately after welding with the nominal model, the CAD model, according to two strategies: best-fit (Figure 12a) and 3-2-1 (Figure 12b).

Figure 12 presents a colorful deviation map as an illustration of the comparison between the geometry of frame 2 right after welding and the corresponding CAD model. Based on the best-fit alignment, a colorful map of deviations is presented, from which it can be observed that—as in the case of frame 1—there are two crucial points on the front surface, where the deviations take the largest values. In contrast, there is concavity in the central part of the frame.

Based on the 3-2-1 alignment (Figure 12b), the front surface deviations indicate a spread of 0.5 mm greater than that of the best-fit alignment, i.e., 3.9 mm. The location of the point with the largest deviation of the frame face coincides with that obtained by the previous method.Similarly, the location of the concavity at the top of the frame coincides in both cases. The longest beam on the side surface has a characteristic convexity that is 1.32 mm, which in the next stage of the analysis has a decisive influence on the design of the datum system and the torsion of the component model relative to CAD. The mounting posts are offset by about 2.5 mm in this setup. The center beam of the frame parallel to the longest beam is offset by nearly 3 mm. During further analysis and subsequent assembly, this suggests that these are the aspects to focus on.

### 3.6. Comparison of Frame 2’s Geometry after Vibratory Stress Relief with the CAD Model

**Figure 13:**
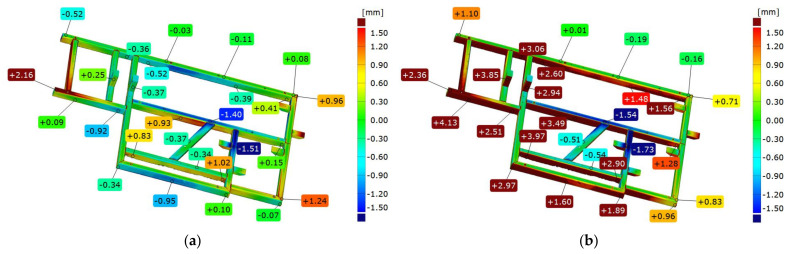
Example of comparison of frame 2 geometry immediately after vibratory stress relief with the CAD model according to two strategies: best-fit (**a**) and 3-2-1 (**b**).

After vibratory stress relief, the frame was scanned again, and result was compared with the CAD model. The colorful deviation map determined from the best-fit (Figure 13a) shows that locations of the extreme deviation are situated in the same places as before relaxation. However, their values change. On the front surface, the spread of values is 3.67 mm, an increase of nearly 0.3 mm. The position of the mounting posts also changed by about 0.3 mm. The longest frame beam on the side surface has a characteristic convexity that is 1.32 mm, which has a decisive impact on the design of the datum system in the next stage of the analysis.

In the 3-2-1 alignment (Figure 13b), the axis of the system runs along the longest beam of the frame, and the aforementioned convexity determines the twist of the model measured against the CAD. This results in a deepening of the tendencies noted during the analysis of frame 2 scanned immediately after welding. The mounting posts are shifted by 3.85 mm and 2.94 mm, respectively, and the middle beam parallel to the longest one is shifted by 3.49 mm; these are definitely higher values than those obtained directly after welding.

### 3.7. Comparison of Frame 2’s Geometry Immediately after Welding and after Vibratory Stress Relief

**Figure 14:**
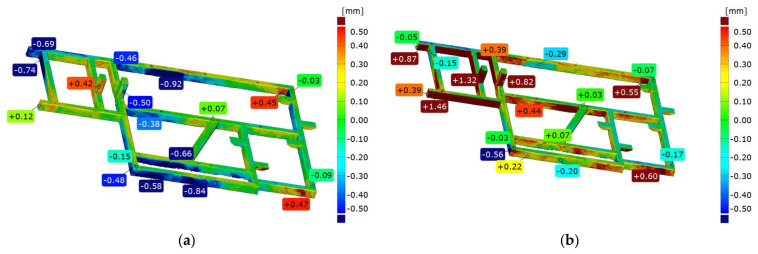
Illustration depicting the contrast between the geometry of frame 1 right after welding and its state following a period of vibratory stress relief according to two strategies: best-fit (**a**) and 3-2-1 (**b**).

Comparing frame 2 scanned directly after welding and after vibratory stress relief, aligning was performed according to the best-fit and 3-2-1 strategies. Figure 14 shows an example of a colorful deviation map for the comparison of frame 1’s geometry immediately after welding and after vibratory stress relief.

The frame directly after welding was established as the reference. In the best-fit alignment, a slight range of deviations can be seen on the front surface. In all other parts of the frame, the spread is 1.39 mm. The convexity of the side surface of the longest frame beam increased by 0.92 mm. On the opposite parallel beam, there was a nonlinear deformation consisting of a bidirectional displacement of one node by −0.48 mm in one direction and −0.58 mm in the other direction. The mounting pillars also shifted after stress release, the first one by +0.42 mm and the second one by −0.50 mm. On the basis of the best-fit alignment, a very complex manner of the element deformation due to stress release is visible. On the longest beam, a “rippling” of the lateral surface is visible, where deviations successively increase, decrease, increase, and then decrease. The extreme longitudinal beam is displaced in the positive direction by 0.63 mm, while the central beam is displaced in the opposite direction by −0.69 mm, which increases their mutual distance by 1.32 mm. The frontal cross-section shows that about halfway through the frame, there is a shift of the frame in the negative direction by an average of 0.6 mm.

The 3-2-1 alignment also shows a slight change in flatness on the face. Determining the axis of the fit along the longest edge, where the so-called waviness was noted earlier, results in the consequent displacement of the remaining beams relative to the layout. The mounting pillars are shifted by 1.32 mm and 0.82 mm, which are significantly higher values than those obtained from previous analyses. Similarly, the deformation of the frame in the extreme area of the longest beam is 50% higher than the corresponding area on the comparison of frames after free relaxation, suggesting that the process of free relaxation should be carried out longer to release all accumulated stresses and finish the deformation of the frame.

### 3.8. Analysis of the Geometric Features of a Welded Frame (Frame 3) for Different Support Methods

The geometry analysis, as for frames 1 and 2, was performed in two coordinate systems. The first is based on aligning the mesh of measured element with a nominal spatial model in the form of a CAD model, according to the best-fit method, while the second is based on alignment according to the 3-2-1 method based on substitute geometric elements. Both approaches present colorful maps of deviations based on alignment with the CAD model as well as cross-sections on three axes showing deformation of the component.

To evaluate the impact of the support point location strategy, a key aspect is the analysis of cross-sections in the *X* and *Y* axes as well as the flatness of the frame face. Table 2 shows extreme values of deviations (max, min), range, and flatness of the front surface for each support variant and both alignment strategies 

The nature of the distribution of deviations for the front surface does not generally change using different support strategies. The locations of extreme deviations are constant, i.e., the minimum value is located in the central part of the central crossbeam, while the maximum value is at one of the beam’s corners (the one located at the beginning of the coordinate system). Only in the case of strategy 14 (i.e., four-point support distributed at the corners of the frame) was there a change in this trend. Then, the level of the beam in the *Y*-axis of the system was aligned in best-fit, and for the 3-2-1 method, the deviation difference in this axis was 0.05 mm.

In each considered case, convexity on the diagonal beam and concavity of the beams parallel to the *X*-axis in the center of the frame were observed. No support method reversed this trend; even concentrating supports in the central part of the frame (variants 10, 12, and 13) did not cause significant deformation of the frame.

For best-fit alignment, the spread of maximum deviations was 0.15 mm; for minimum deviations, it was 0.09 mm; and for total spread, it was 0.15 mm. For 3-2-1 alignment, the spread of maximum deviations was 0.05 mm; for minimum deviations, it was 0.07 mm; and for total spread, it was 0.09 mm.

Comparing the values of boundary deviations and range for the tested spacing strategies of supports, it can be observed that depending on the aligning used, the absolute values of boundary deviations are symmetrically distributed. Larger spreads were obtained for the best-fit method. However, the difference in the spreads did not exceed 0.1 mm for any strategy, as shown in Table 2.

## 4. Conclusions

This paper presents a comprehensive analysis of geometric properties related to welded frames that serve as integral components within a prototype packaging machine. Frames were subjected to two distinct stress relaxation processes that aimed to mitigate welding-induced stresses: free relaxation and accelerated vibratory stress relief. Geometric measurements of the tested frames were meticulously carried out using a photogrammetric system, with temperature control ensuring accuracy within a range of ±1 °C.

Based on the research and analysis, the following conclusions can be drawn:

1. The comparative analysis of the geometric deviations of the frames after free relaxation and vibration relaxation led to confirmation of the assumption that the dimensions of the frame subjected to vibration relaxation deviate more from the nominal dimensions than those of the second case.
2. However, it was noted that frame subjected to vibratory stress relief did not undergo further deformation, which is a desirable outcome compared to the random dimensional changes observed in frames undergoing free relaxation. These random changes can generate problems during assembly with other components, particularly with regard to the proper fit of mounting holes.
3. The metrological analysis evaluating changes in the geometry of the frames supported by fifteen variants of steel frame support highlighted the consistent nature of deviations across different support strategies, affirming the appropriate selection of structural elements to ensure frame stiffness.
4. The range of deformations across of the longest beam was 50% higher than on the corresponding area on the frames after free relaxation, which suggests that the process of free relaxation should be carried out for longer to release all accumulated stresses and finish the deformation of the frame. However, it is not certain that an extension of the free relaxation time would maintain this deformation trend.

In summary, the application of accelerated vibratory stress relief effectively controls spontaneous dimensional changes in the designed frames of the packaging machine that result from welding-induced stresses. This approach offers advantages over free relaxation, particularly in terms of shortening the relaxation process.

## Figures and Tables

**Table 1:** Summary of parameters of sequential vibratory stress relief.

Sequence	Rotational Speed [rpm]	Frequency [Hz]	Time [min]	Centrifugal Force [kN]	Direction of Response
1	1896.0	31.60	7.0	0.29803	z
2	3057.6	50.96	6.5	0.77507	y
3	4836.0	80.60	6.0	1.93888	z
4	6298.8	104.98	4.0	3.28923	y
5	7781.4	129.69	3.5	5.01989	y
6	8511.6	141.86	4.0	6.00622	z
7	8699.4	144.99	5.5	6.27419	z

**Table 2:** Summary of max, min, range and flatness deviation values for the frame No. 3.

Option	Alignment	Max [mm]	Min [mm]	Range [mm]	Flatness [mm]
1	Best-fit	0.31	−0.21	0.52	1.19
	3-2-1	0.05	−0.41	0.46	
2	Best-fit	0.23	−0.26	0.49	1.18
	3-2-1	0.02	−0.44	0.46	
3	Best-fit	0.30	−0.22	0.52	1.04
	3-2-1	0.03	−0.40	0.43	
4	Best-fit	0.30	−0.21	0.51	1.44
	3-2-1	0.04	−0.43	0.47	
5	Best-fit	0.30	−0.20	0.50	1.18
	3-2-1	0.05	−0.40	0.45	
6	Best-fit	0.27	−0.19	0.46	1.11
	3-2-1	0.03	−0.37	0.40	
7	Best-fit	0.30	−0.22	0.52	1.15
	3-2-1	0.06	−0.39	0.45	
8	Best-fit	0.31	−0.20	0.51	1.25
	3-2-1	0.07	−0.40	0.47	
9	Best-fit	0.30	−0.24	0.54	1.47
	3-2-1	0.04	−0.44	0.48	
10	Best-fit	0.31	−0.24	0.55	1.41
	3-2-1	0.06	−0.39	0.45	
11	Best-fit	0.35	−0.20	0.55	1.14
	3-2-1	0.06	−0.43	0.49	
12	Best-fit	0.33	−0.19	0.52	1.32
	3-2-1	0.07	−0.37	0.44	
13	Best-fit	0.31	−0.18	0.49	1.47
	3-2-1	0.02	−0.40	0.42	
14	Best-fit	0.20	−0.20	0.40	1.22
	3-2-1	0.06	−0.42	0.48	
15	Best-fit	0.31	−0.17	0.48	1.24
	3-2-1	0.03	−0.37	0.40	

## Data Availability

The data presented in this study are available on request from the corresponding author.
